# Characterization of Differentially Abundant Proteins Among *Leishmania (Viannia) braziliensis* Strains Isolated From Atypical or Typical Lesions

**DOI:** 10.3389/fcimb.2022.824968

**Published:** 2022-02-15

**Authors:** Bárbara B. Esteves, Marcella N. Melo-Braga, Vladimir Gorshkov, Thiago Verano-Braga, Martin R. Larsen, Célia M. F. Gontijo, Patricia F. Quaresma, Helida M. Andrade

**Affiliations:** ^1^ Laboratório de Leishmanioses, Departamento de Parasitologia, Instituto de Ciências Biológicas, Universidade Federal de Minas Gerais, Belo Horizonte, Brazil; ^2^ Laboratório de Biologia Sintética e Biomiméticos, Departamento de Bioquímica e Imunologia, Instituto de Ciências Biológicas, Universidade Federal de Minas Gerais, Belo Horizonte, Brazil; ^3^ Department of Biochemistry and Molecular Biology, University of Southern Denmark, Odense, Denmark; ^4^ Núcleo de Proteômica Funcional, Departamento de Fisiologia e Biofísica, Instituto de Ciências Biológicas, Universidade Federal de Minas Gerais, Belo Horizonte, Brazil; ^5^ Study Group in Leishmaniosis, Instituto René Rachou (IRR) –Fundação Oswaldo Cruz (FIOCRUZ/MG) Belo Horizonte, Belo Horizonte, Brazil; ^6^ Departamento de Microbiologia Imunologia e Parasitologia, Universidade Federal de Santa Catarina, Florianópolis, Brazil

**Keywords:** *Leishmania braziliensis*, atypical wounds, proteome, abundance, TMT, antimony resistance

## Abstract

*Leishmania (Viannia) braziliensis* is the main etiological agent of cutaneous and mucocutaneous leishmaniasis in Latin America. Non-ulcerated atypical tegumentary leishmaniasis cases caused by *L. braziliensis* have been reported in several regions of the American continent, including the Xacriabá indigenous reserve in São João das Missões/Minas Gerais, Brazil. Parasites isolated from these atypical clinical lesions are resistant to antimony-based therapeutics. In the present study, proteins displaying differential abundance in two strains of *L. braziliensis* isolated from patients with atypical lesions compared with four strains isolated from patients with typical lesions were identified using a quantitative proteomics approach based on tandem mass tag labeling (TMT) and mass spectrometry. A total of 532 (*P*<0.05) differentially abundant proteins were identified (298 upregulated and 234 downregulated) in strains from atypical lesions compared to strains from typical lesions. Prominent positively regulated proteins in atypical strains included those that may confer greater survival inside macrophages, proteins related to antimony resistance, and proteins associated with higher peroxidase activity. Additionally, we identified proteins showing potential as new drug and vaccine targets. Our findings contribute to the characterization of these intriguing *L. braziliensis* strains and provide a novel perspective on Atypical Cutaneous Leishmaniasis (ACL) cases that have been associated with therapeutic failures.

## 1 Introduction

In different parts of the world, authors have referred to Atypical Cutaneous Leishmaniasis (ACL) cases as sporotrichoid, reported in Pakistan, but no *Leishmania* species was identified (Iftikhar et al., 2003), erysipeloid, reported in Anatolia, Turkey, also unidentified species of *Leishmania* (Karincaoglu et al., 2004), recidiva cutis, caused by *L. panamensis* (Calvopina et al., 2005) or zosteriform, reported in Iran (Omidian and Mapar, 2006). On the other hand, *L eishmania (Viannia) braziliensis* is an important *Leishmania* species that circulates in several North and South American countries. This species can cause a wide spectrum of clinical manifestations ranging from single and typical lesions with a granular background and raised edges, known as cutaneous leishmaniasis (CL), to the involvement of nasal and oral mucous membranes, also known as the cutaneous-mucosal or mucosal form (Lainson and Shaw, 1998). The spectrum of injuries caused by *L. braziliensis* also includes reports of atypical cutaneous leishmaniasis (ACL), with characteristics such as papules, verrucous and keloid lesions, and crusted and ulcerated plaques, distinct from those presented by common injuries (Baptista et al., 2009; Guimarães et al., 2009; Quaresma et al., 2018). In an area hyperendemic for *L. braziliensis* transmission in northeastern Brazil, ACL accounted for 1.9% of all tegumentary leishmaniasis cases (Guimarães et al., 2009). Possible causes associated with ACL are host immune status (pregnancy, co-morbidities, immunosuppression (Iftikhar et al., 2003; Omidian and Mapar, 2006), environmental factors, and strain of the parasite (Guimarães et al., 2016). However, the majority of such patients do not present with clinical co-morbidities or HIV infection and are not using immunosuppressive drugs (Da-Cruz et al., 1999). Furthermore, *L. braziliensis* is characterized by variable infectivity, virulence, and response to anti-leishmanial therapy (Rego et al., 2018; Patino et al., 2019a); thus, there is no consensus on the factors involved in cases of ACL, although these data seem to suggest that parasite factors may be more determinant.

In Brazil, ACL cases have been reported in the states of Rio de Janeiro (Baptista et al., 2009), Bahia (Guimarães et al., 2009), and Minas Gerais (Quaresma et al., 2018) and are associated with genetic variations of the parasite and lower response to treatment with antimonials. In the state of Minas Gerais (MG), ACL cases were observed in patients from the Xacriabá indigenous reserve located in the municipality of São João das Missões. The reserve is predominantly composed of Brazilian savannah (cerrado) vegetation, and *Lutzomyia intermedia* is the main vector of *L. (V.) braziliensis* in this region (Rego et al., 2018). In patients from Xacriabá, cytokine/chemokine expression in skin biopsies is associated with the time of lesions, but not with the type of injury. Additionally, histopathological analysis of cutaneous tissue samples did not show any differences between patients with different types of lesions (Costa-Silva et al., 2014). However, genetic polymorphisms are correlated with typical or atypical clinical manifestations (Quaresma et al., 2018). A large number of therapeutic failures were observed in patients with atypical lesions, and an *in vitro* study of antileishmanial drug susceptibility reported that strains from atypical manifestations are more resistant to Glucantime, the antileishmanial treatment recommended by the WHO (Rugani et al., 2019). Moreover, atypical manifestations have been demonstrated to be related to therapeutic failure (Guimaraes et al., 2016).

Proteomic analysis is a fundamental tool for understanding physiological processes and for studying the relationships between the genotype and the phenotype. Quantitative proteomics have been used for several *Leishmania* species in studies aiming at different aspects of biology, such as identifying protein modification during differentiation in *L. donovani* (Pawar et al., 2020), identification of differentially abundant proteins probably involved in the virulence of amastigote and promastigote forms of *L. infantum* (Fialho-Junior et al., 2021), and analysis of *in vitro* and *in vivo* models of cutaneous leishmaniasis using *L. amazonensis* and *L. major* (Negrão et al., 2019). Here, we performed a proteomic analysis using TMT labeling followed by mass spectrometry (MS) to identify differentially abundant proteins in *L. braziliensis* promastigotes that cause typical and atypical lesions. Our findings contribute to the characterization of the selected *L. braziliensis* strains, highlighting the importance of conducting new studies to elucidate the factors involved in the wide diversity of clinical manifestations and drug response outcomes in clinical practice.

## 2 Materials And Methods

### 2.1 Ethics Statement

*Leishmania* field strains were obtained from human patients living in the Xacriabá indigenous community located in São João das Missões municipality, Minas Gerais, Brazil (Quaresma et al., 2018). Samples from other endemic areas were obtained from the outpatient care clinic at Centro de Referência em Leishmanioses – Instituto René Rachou-IRR/FIOCRUZ from 1993 to 1998. All patients involved in this study provided written informed consent, and experimental procedures were approved by the IRR Research Ethics Committee in Human Research, the National Committee for Research Ethics (Comissão Nacional de Ética em Pesquisa − CONEP; process 355/2008), and the National Indian Foundation (Fundação Nacional do Índio – FUNAI; n° 149/CGEP/08).

### 2.2 Parasites

The *L. braziliensis* strains from typical (LbTL) lesions were isolated from patients from Belo Horizonte, MG, Brazil, identified as MHOM/BR/95/RR051 and BH17 and from São João das Missões, MG, Brazil identified as MHOM/BR/08/426 and MHOM/BR/09/346. The strains from atypical lesions (LbAL) were isolated from São João das Missões, MG, Brazil, identified as MHOM/BR/08/316 and MHOM/BR/08/340. The clinical features of typical and atypical lesions from which the parasites were isolated and the isolation and confirmation of the species were performed as described by Quaresma et al. (2018). In summary, the typical lesions were characterized like circular or oval ulcers with an erythematous, infiltrated, firm, reddish and granular base and a well-delimited, elevated border. Atypical lesions were defined by the presence of unusual cutaneous wounds.

The isolation procedures were accomplished added lesion fragments to tubes containing NNN medium (Novy and McNeal, 1903; Nicolle, 1908) enriched with liquid Schneider’s medium and incubated at 25 ± 1°C. The cultures were examined weekly and were considered positive when *Leishmania* promastigotes were observed. The species identification methods performed for characterizing the isolates used in this study was done by the *Leishmania* Isolation, Culture and Typing Service of the Leishmania Collection of the Oswaldo Cruz Institute (Coleção de Leishmania do Instituto Oswaldo Cruz – CLIOC) according to the protocol described by Cupolillo et al. (1994). We also used hsp70-PCR-RFLP which amplified a 1300-bp fragment of the hsp70 gene and subsequently the restriction profiles were obtained from HaeIII digestion of the PCR products. In addition to these methods the sequencing of the 1,300 bp fragment of hsp70 were performed. The combination of identification methods allowed the characterization of parasites as *L. braziliensis*.

Promastigotes of *L. braziliensis* were maintained in M199 culture media (SigmaAldrich/Merck) supplemented with 10% fetal bovine serum (ThermoFischer/Gibco), 100 units/mL penicillin (ThermoFischer/Gibco), 100 μg/mL streptomycin (ThermoFischer/Gibco), 12.5 mM glutamine (ThermoFischer/Gibco), 0.1 M adenine (SigmaAldrich/Merck), 205 μg/mL hemin, and 40 mM HEPES (SigmaAldrich/Merck), pH 7.4 at 26°C. Overall, six isolates were evaluated: four from typical lesions and two from atypical lesions. All strains were cultivated in independent triplicates under identical conditions until the 9th passage, when promastigotes were obtained in the final stage of the stationary growth phase. This phase was established after performing the growth curve for eight consecutive days. During this period, motility and morphological aspects such as cell body and flagellum length were also observed. Thus, promastigotes forms were harvested in the stationary phase (5^th^ day) by centrifugation at 3,000 x *g* for 15 min at 4°C. Cell pellets were washed three times in phosphate-buffered saline (PBS), pH 7.2, and frozen at −80°C until use.

### 2.3 Quantitative Proteomics

#### 2.3.1. Protein Extraction and TMT-Labeling

Frozen promastigotes, as described above, (LbAL and LbTL) were lysed for 2 h at 24°C in lysis buffer [8 M urea, 2 M thiourea, 4% CHAPS, 65 mM dithiothreitol (DTT), 1 M Tris, 40 mM protease inhibitor cocktail containing serine protease inhibitors, and cysteine protease inhibitors (Protease Inhibitor Mix; GE Healthcare, USA)] at a density of 500 μL per 10^9^ parasites. The cell lysate was passed 10 times through a 26G needle and centrifuged at 20000 × *g* for 20 min. The supernatant was aliquoted and stored in a freezer at −80°C until use. Protein concentration was determined using a QuantKit 2D (GE Healthcare) according to the manufacturer’s recommendations.

Equal amounts of protein (300 µg) from each sample were reduced *via* incubation with 50 mM dithiothreitol (DTT) for 1 h at 24°C and subsequently alkylated with 150 mM iodoacetamide (IAA) for 30 min at room temperature in the dark. Before the digestion step, the samples were washed with buffer (6M urea and 50 mM TEAB) to dilute the CHAPS present in the lysis buffer. Protein purification and concentration filters (Micon ^®^ Ultra-0.5 mL) were used for these washes. Enzymatic digestion was performed using trypsin (Promega, Madison) at a ratio of 1:50 for 16 h at 37°C.

After protein digestion, peptides were labeled with specific isobars from the TMT^®^ 10plex Isobaric Mass Tagging Kit (Thermo Fisher Scientific/Pierce Biotechnology, Rockford, USA) following the manufacturer’s instructions. Briefly, 15 μg of each sample was labelled with 0.266 mg of an isobaric amine-reactive tag (TMT10-126, TMT10-127N, TMT10-127C, TMT10-128N, TMT10-128C, and TMT10-129N). The reaction mixture was incubated for one hour at room temperature for complete labeling. The reaction was quenched by the addition of 5% hydroxylamine, and labelled peptides were combined at a ratio of 1:1:1:1:1:1. The samples were dried using a SpeedVac and stored for further analysis. The preparation process was performed in biological triplicates.

#### 2.3.2 High pH Reversed Phase Pre-Fractionation

Labeled peptide mixtures were dissolved in buffer A (20 mM ammonium formate, pH 9.3) and fractioned using an Acquity UPLC M-Class Peptide CSH C18 column (1.7 μm, 300 μm x 100 mm) (Waters) on an UltiMate 3000 high-pressure liquid chromatography (HPLC) system (Dionex, Sunnyvale, CA, USA) operating at 5 μL/min. Each set was eluted using the following chromatographic gradient expressed as a percentage of solvent B (20% solution A/80%ACN): 2–40% for 27 min, 40–50% in 4 min, 50–70% in 4 min, and 70–95% in 5 min. Each sample was separated into 15 fractions that were further merged into five concatenated fractions, and dried using a SpeedVac prior to MS analysis.

#### 2.3.3 LC-MS/MS

Pre-fractionated TMT-labeled peptides were resuspended in solvent A (0.1% (v/v) formic acid) and subsequently injected into an EASY-nLC system (Thermo) with a two-column system setup. The pre-column was 3 cm in length and 100 µm in inner diameter, and it was packed with 5 μm (particle size) Reprosil-Pur C18-AQ resin (Dr. Maisch GmbH). An analytical column (17 cm × 75 µm) was packed with a 3 μm Reprosil-Pur C18-AQ resin (Dr. Maisch GmbH). Peptides from each fraction were eluted for 68 min using the following gradient expressed as a percentage of solution B (95% acetonitrile/0.1% formic acid): 2–5% in 1 min, 5–25% in 50 min, 25–40% in 10 min, 40–95% in 1 min, 95% in 5 min and 95–2% in 1 min, at a constant flow of 300 nL/min. The Q Exactive HF-X hybrid quadrupole-Orbitrap mass spectrometer (Thermo) was operated in positive mode using data-dependent acquisition (DDA) (Top 20). Peptide ions were resolved in the Orbitrap (MS), in the range of 350 to 1500 m/z with a resolution of 120,000 FWHM (AGC target 3e6 or maximum injection time of 50ms). At each MS, the 20 most intense ions (Top 20), with the minimum AGC target 1e3, were selected in the quadrupole, using an isolation window equal to 0.7 m/z. These ions were selected for fragmentation (MS2) by higher-energy collision dissociation (HCD), with a normalized collision energy of 33%. The originating fragments were resolved in Orbitrap with a resolution of 45,000 FWHM (AGC target 1e5 or maximum injection time of 60 ms) and included in a dynamic exclusion list for 30 s.

#### 2.3.4 Data Analysis

Raw data were analyzed using the Proteome Discoverer 2.1 software. The database search was performed by the SEQUEST using the *Leishmania braziliensis* NCBI database (25,611 sequences). Database search parameters included: i) maximum of two trypsin missing cleavages, ii) carbamidomethylation of cysteine ​​as a fixed modification, iii) oxidation of methionine, N-terminal acetylation of protein, and TMT 6plex (lysine and N-terminal peptide) as variable modifications, iv) precursor and fragment mass tolerance of 10 ppm and 0.02 Da, respectively; v) 1% false discovery rate (FDR); and vi) unique peptides plus razor ones were grouped into the respective proteins. Only master proteins with a 1% FDR were considered for the analysis.

Before statistical analyses, we normalized the log2 transformed ion reporter abundance using the “control” sample (pool of all analyzed samples) for all three datasets. Subsequently, data were normalized by subtracting the median of each group using the Perseus program (https://maxquant.net/perseus). After combining the three sets, we subtracted the average over all conditions (biological/technical) for each protein. Proteins significantly regulated between LbAL and LbTL conditions were determined by the one-way ANOVA statistical test (*P*< 0.05) plus log2 fold change of 0.584, using DanteR software (Polpitiya et al., 2008).

The differentially abundant proteins were functionally categorized through annotations of biological processes (BP) and molecular functions (MF) cataloged according to the Gene Ontology (GO) consortium. This set of genomic data was analyzed using the bioinformatics platform TriTrypDB release 40 (http://tritrypdb.org/tritrypdb/). For each category, Fischer’s exact test was used to test the enrichment of selected proteins against all proteins identified in the study. A false discovery rate (FDR) and an adjusted *P*< 0.05 were set as thresholds to define the significance of the functional enrichment.

To evaluate the potential relationship among these proteins using enriched GO categories, we predicted the protein-protein interaction networks associated with the whole proteome of the organism. For this prediction analysis, we used the STRING 10.5 database with a cut-off value of 0.4 considered to be of medium confidence. This database provides a network of predicted interactions or interactions already described in the literature between proteins or between genes (protein-protein interaction networks, http://string-db.org). Finally, the KEGG (Kyoto Encyclopedia of Genes and Genomes) database was used *via* the metabolic pathways enrichment tool (TritrypDB bioinformatics platform) to analyze the pathways of the identified differentially abundant proteins and to understand their biological significance.

#### 2.3.5 Data Availability Statement

The mass spectrometry proteomics data have been deposited to the ProteomeXchange Consortium *via* the PRIDE partner repository with the dataset identifier PXD029995.

### 2.4 Validation Proteomic Data

#### 2.4.1 Peroxidase Activity

Peroxidase activity was measured using 10^8^ promastigotes from each strain in the LbTL and LbAT groups. Promastigotes were lysed by gentle shaking in 50 μL of 0.1% Triton X-100 for 30 min at room temperature. Next, 50 μM H_2_O_2_ (final concentration) was added, followed by incubation at 37°C for 30 min. The reaction was stopped by adding 0.75 mL of trichloroacetic acid solution (8% v/v), and the precipitate was removed by centrifugation. To the supernatant, 0.2 mL of 10 mM ferrous ammonium sulphate and 0.1 mL of 2.5 M potassium thiocyanate were added, and the remaining amount of H_2_O_2_ was determined spectrophotometrically (480 nm) by calibration against a standard curve with known concentrations of H_2_O_2_ (Jiang et al., 2005).

Statistical analyses were performed using GraphPad Prism version 5.00 for Windows (GraphPad Software, San Diego, CA, USA). Data are presented as means of the group. Comparisons between different groups were made using one-way ANOVA followed by Bonferroni’s test, and statistical significance was set at *P*< 0.05.

## 3 Results

### 3.1 Identification and Relative Abundance of Proteins From Atypical and Typical Strains

**Figure 1:**
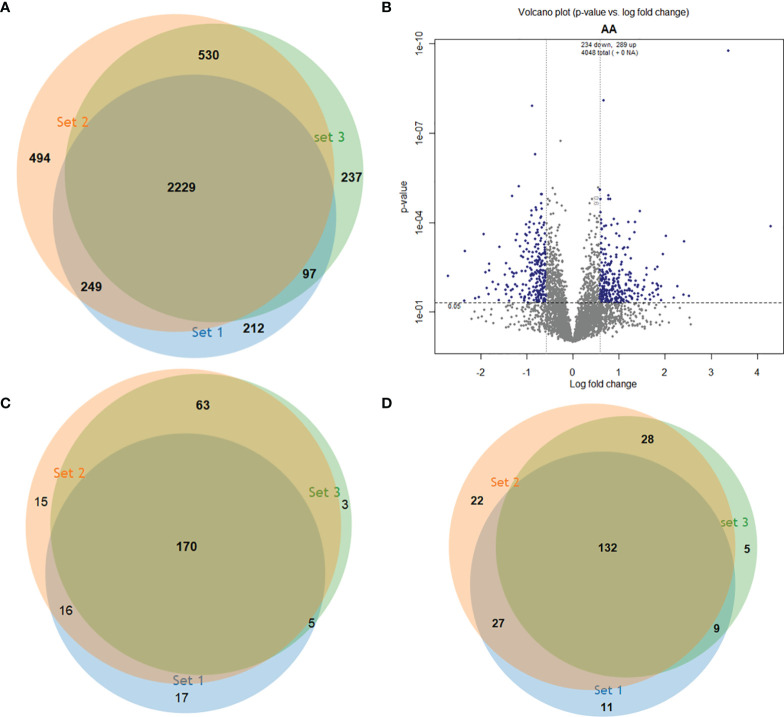
Identification of differentially abundant proteins in three experimental replicates. **(A)** Venn diagram showing the 4,048 proteins identified in the 3 independent runs. **(B)** Volcano plot of proteins with statistically significant changes in abundance after ANOVA (*P*<0.05) (y axis) and fold change in log2 of 0.584 (1.5) (x axis). **(C)** Upregulated proteins in each run. **(D)** Downregulated proteins in each run.

We performed an isobaric tagging and high-resolution mass spectrometry strategy to compare the proteome of *L. braziliensis* strains that cause atypical lesions (LbAL, n=2) and *L. braziliensis* strains that cause typical lesions (LbTL, n=4). Each strain was analyzed in triplicate, resulting in triplicates of independent experiments. This approach enabled the identification of 4,048 non-redundant proteins (**Supplementary Table S1**), of which 2,229 proteins were present in all three independent runs (**Figure 1A**). To assess the differentially abundant proteins, we compared the relative abundance values of the identified proteins in LbAL *versus* LbTL strains. Our results showed that the abundance of 298 and 234 proteins significantly increased and decreased in LbAL strains, respectively, compared to LbTL strains (**Figure 1B**). However, when we considered only proteins identified in at least two of the three runs, 254 (**Supplementary Table S2**) and 196 (**Supplementary Table S3**) proteins were upregulated and downregulated at LbAL *versus* LbTL (**Figures 1C, D**).

### 3.2 Functional Analysis

**Figure 2:**
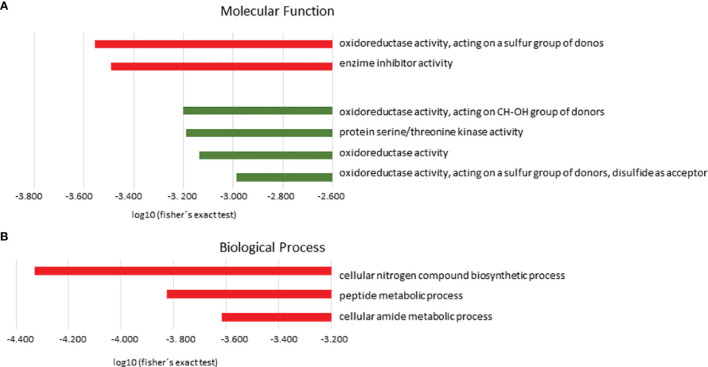
Gene ontology enrichment analysis (GO). Protein distribution based on molecular functions **(A)** and biological processes **(B)** predicted in *L. braziliensis* from the atypical lesions group in relation to the typical lesions group. In red, categories enriched in the upregulated proteins group; in green, categories enriched in the downregulated proteins group. Terms overrepresented by *P*< 0.05 in Fisher’s exact test, filtering for FDR < 0.05.

Gene Ontology (GO) data were used to glean insights into the differences between atypical and typical *L. braziliesnis* strains. The GO enrichment of regulated proteins associated GO term to 37 proteins with increased abundance and 16 with decreased abundance in atypical strains compared to typical strains. These proteins were categorized into functional groups (**Figure 2**).

Regarding Molecular Function (MF), the category Oxidoreductase activity, acting on a sulfur group of donors (*P*=4.11e-4) followed by enzyme inhibitor activity (*P*=3.33e-4) were significantly enriched in the group of upregulated proteins. These groups are represented mainly by the following proteins: trypanothione reductase, peptide methionine sulfoxide reductase-like, and ecotin. In the group of downregulated proteins, we observed that oxidoreductase activity, acting on CH-OH group of donors (6.27e-4), protein serine/threonine kinase activity (*P*=6.45e-4), oxidoreductase activity (*P*=7.30e-4), and oxidoreductase activity, acting on a sulfur group of donors, disulfide as acceptor (*P*=1.03e-3) were among the significant categories. These groups are represented mainly by the following proteins: malate dehydrogenase and choline dehydrogenase (**Figure 2A** and **Table 1**).

**Table 1:** Gene Ontology (GO) enrichment analysis of Molecular Function in differentially regulated proteins in *L. braziliensis* from atypical lesion (LbAL) compared to *L. braziliensis* from typical lesion (LbTL).

TERM	Fold change (P value)	PROTEINS	RATIO (LbAL vs LbTL)
**Upregulated proteins**			
*Oxidoreductase activity, acting on a sulfur group of donors*	7.43 (4.11e-4)	trypanothione reductase	2.417
		peptide_methionine sulfoxide reductase-like	1.060
		putative thioredoxin	0.984
		thiol-dependent_reductase_1	0.709
		putative glutaredoxin-like protein	0.649
*Enzime inhibitor activity*	18.26 (3.33e-4)	ecotin	1.917
		Ecotin-like protein 2	0.797
		Protein phosphatase inhibitor 2 (IPP-2)	0.768
***Downregulated proteins***			
*Oxidoreductase activity. acting on CH-OH group of donors*	7.02 (6.27e-4)	mitochondrial malate dehydrogenase	-1.150
		choline dehydrogenase like protein,	-1.062
		glycosomal malate dehydrogenase	-0.944
		dehydrogenase-like protein	-0.888
		putative short chain 3-hydroxyacyl-CoA dehydrogenase	-0.825
*Protein serine/threonine kinase activity*	9.70 (6.45e-4)	putative target of rapamycin (TOR) kinase 1	-1.744
		putative protein kinase	-1.029
		protein kinase A catalytic subunit isoform 1	-0.765
		protein kinase A catalytic subunit	-0.676
*Oxidoreductase activity*	2.73 (7.30e-4)	mitochondrial malate dehydrogenase	-1.150
		choline dehydrogenase, like protein	-1.062
		putative short chain dehydrogenase	-1.017
		conserved hypothetical protein (134065654)	-0.978
		glycosomal malate dehydrogenase	-0.944
		putative endoplasmic reticulum oxidoreductin	-0.938
		dehydrogenase-like protein	-0.888
		putative short chain 3-hydroxyacyl-CoA dehydrogenase	-0.825
		fatty acid desaturase	-0.700
		aldehyde dehydrogenase, mitochondrial precursor	-0.696
		conserved hypothetical protein (134063632)	-0.662
		putative C-1-tetrahydrofolate synthase, cytoplasmic	-0.655
*Oxidoreductase activity, acting on a sulfur group of donors, disulfide as acceptor*	35.57 (1.03e-3)	putative endoplasmic reticulum oxidoreductin	-0.938
		conserved hypothetical protein (134063632)	-0.662

Proteins sorted by log2-ratio.

The biological process (BP) with statistical significance among proteins with increased abundance in atypical strains compared to typical strains were cellular nitrogen compound biosynthetic process (P=4.68e-5 with 27 proteins), peptide metabolic process (P=1.49e-4 with 20 proteins), and cellular amide metabolic process (P=2.42e-4 with 20 proteins). These groups are mainly represented by ribosomal proteins. In contrast, no significantly enriched category was found for downregulated proteins (**Figure 2B** and **Table 2**)

**Table 2:** Gene Ontology (GO) enrichment analysis of Biological Process in differentially regulated proteins in *L. braziliensis* from atypical lesion (LbAL) compared to *L. braziliensis*. Proteins sorted by log2-ratio.

TERM	Fold change (P value)	PROTEINS	RATIO (LbAL vs LbTL)
**Upregulated proteins**			
*Cellular nitrogen compound biosynthetic process*	2.22 (4.68e-5)	putative 60S ribosomal protein L10a	1.807
		40S ribosomal protein S24e	1.398
		60S ribosomal protein L32	1.357
		putative DNA-directed RNA polymerase II	1.342
		FtsJ-like_methyltransferase	1.287
		DNA_primase_large_subunit	1.237
		Chain B, Dihydroorotate dehydrogenase	1.224
		nucleoside diphosphate kinase b	1.072
		putative 60S acidic ribosomal protein	1.048
		putative 40S ribosomal protein S30	1.041
		deoxyribose-phosphate_aldolase	0.969
		conserved hypothetical protein	0.954
		kynureninase	0.952
		conserved hypothetical protein	0.891
		60S_acidic_ribosomal_protein_P2	0.816
		inosine-guanosine_transporter	0.770
		acidic ribosomal P2 beta protein	0.769
		putative methylthioadenosine phosphorylase	0.717
		putative 60S ribosomal protein L13a	0.712
		putative 60S ribosomal protein L19	0.708
		putative arginyl-tRNA synthetase	0.669
		putative ribosomal protein S7	0.624
		putative asparaginyl-tRNA synthetase	0.623
		putative ribosomal protein l35a	0.616
		putative ribosomal protein L27	0.609
		putative ribosomal protein L15	0.601
		putative 60S ribosomal protein L5	0.601
***Peptide metabolic process and***	2.47 (1.49e-4)	putative 60s ribosomal protein l10a	1.807
***Cellular amide metabolic process***	2.38 (2.42e-4)	40S ribosomal protein S24e	1.398
		60S ribosomal protein L32	1.357
		FtsJ-like_methyltransferase	1.287
		conserved hypothetical protein	1.119
		putative 60S acidic ribosomal protein	1.048
		putative 40S ribosomal protein S30	1.041
		conserved hypothetical protein	0.891
		60S_acidic_ribosomal_protein_P2	0.816
		acidic ribosomal P2 beta protein	0.769
		putative 60S ribosomal protein L13a	0.712
		thiol-dependent_reductase_1	0.709
		putative 60S ribosomal protein L19	0.708
		putative arginyl-tRNA synthetase	0.669
		putative ribosomal protein S7	0.624
		putative asparaginyl-tRNA synthetase	0.623
		putative ribosomal protein l35a	0.616
		putative ribosomal protein L27	0.609
		putative ribosomal protein L15	0.601
		putative 60S ribosomal protein L5	0.601

### 3.3 Prediction of Protein-Protein Interaction (PPI)

**Figure 3:**
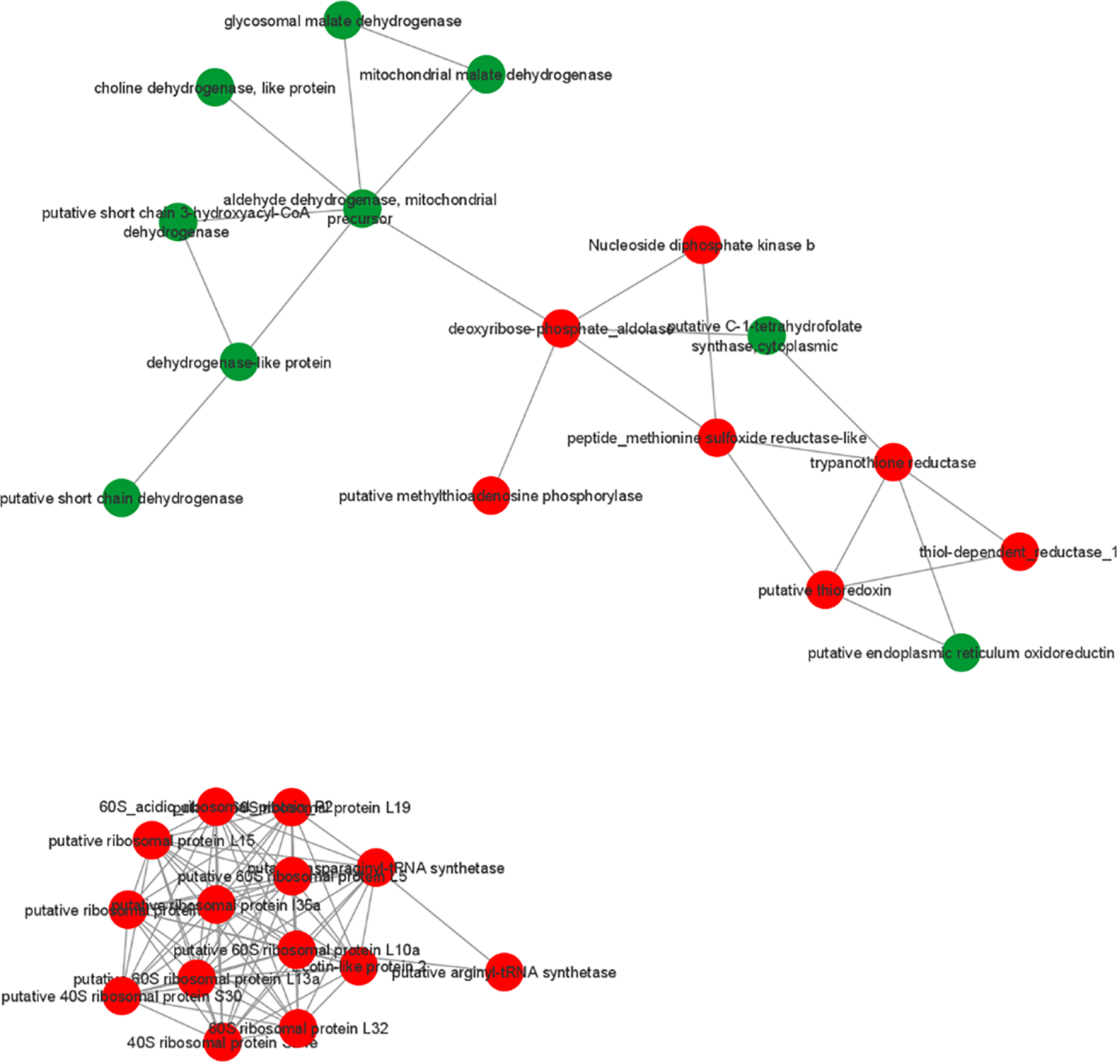
Prediction of interaction among differentially abundant proteins in *L. braziliensis* that are part of enriched GO categories. Red and green represents upregulated and downregulated proteins in LbAL *vs* LbTL comparison.

To identify possible interactions between the identified proteins, a prediction analysis of protein interactions was performed using String 10.5. The network obtained was composed of 21 upregulated and 9 downregulated proteins. The obtained networks were later edited in Cytoscape 3.4.0, to assign colors to positive and negative adjustment values. The positively regulated proteins interacted with each other, mainly represented by reductase proteins, and similarly, the negatively regulated proteins interacted with each other, mainly represented by dehydrogenases proteins. Moreover, it is possible to observe the interactions among ribosomal proteins, all of which are positively regulated in strains isolated from atypical lesions compared to strains isolated from typical lesions (**Figure 3**).

### 3.4 Analysis of Enrichment of Metabolic Pathways

**Figure 4:**
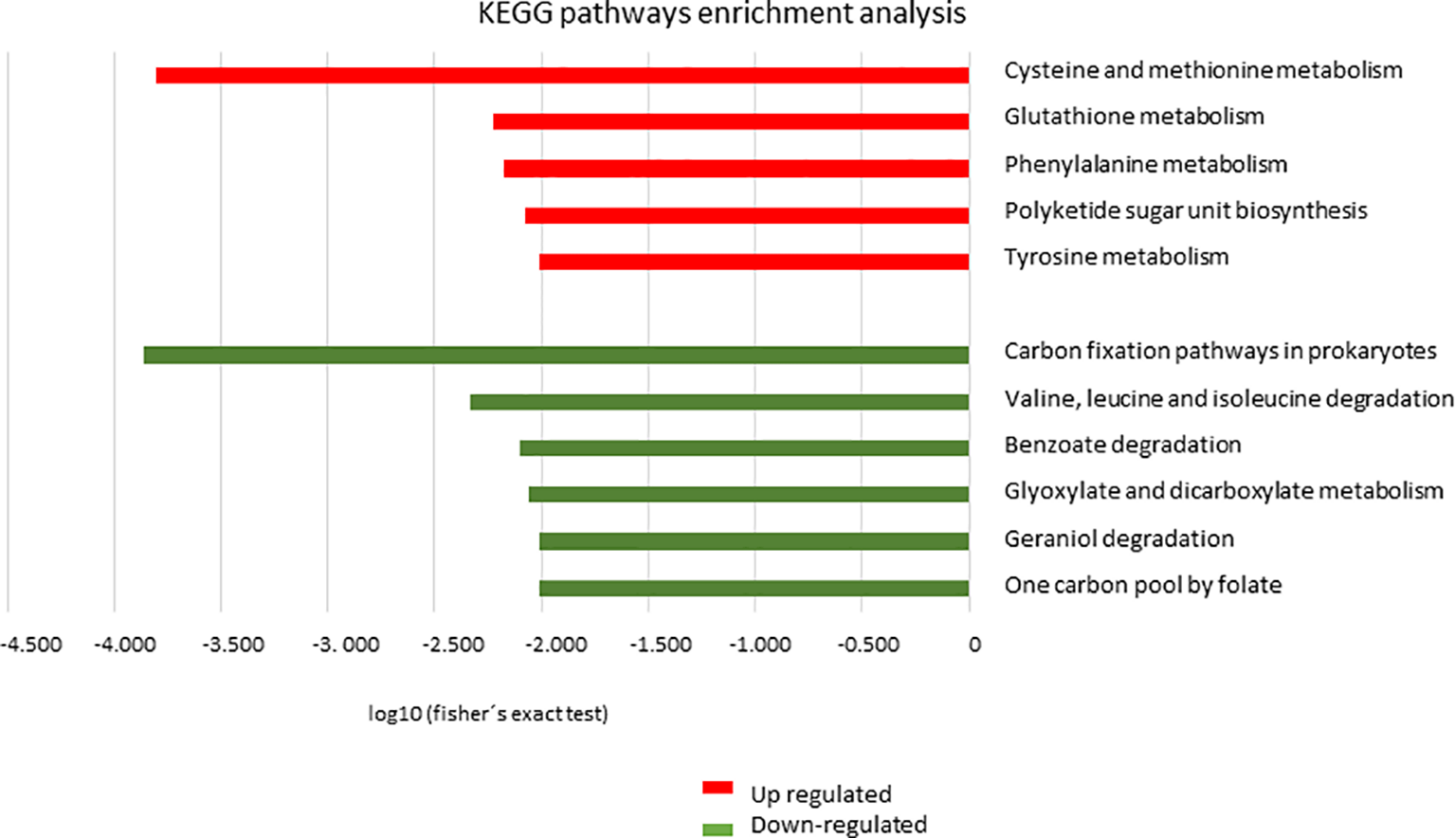
Analysis of enrichment of metabolic pathways by KEGG. Distribution of proteins based on metabolic pathways in *L. braziliensis* from the atypical lesions group (LbAL) compared to the typical lesions group (LbTL). In red, metabolic pathways enriched in the upregulated protein group; in green, metabolic pathways enriched in the downregulated protein group. Terms overrepresented by *P* < 0.01 in Fisher’s exact test.

**Figure 5:**
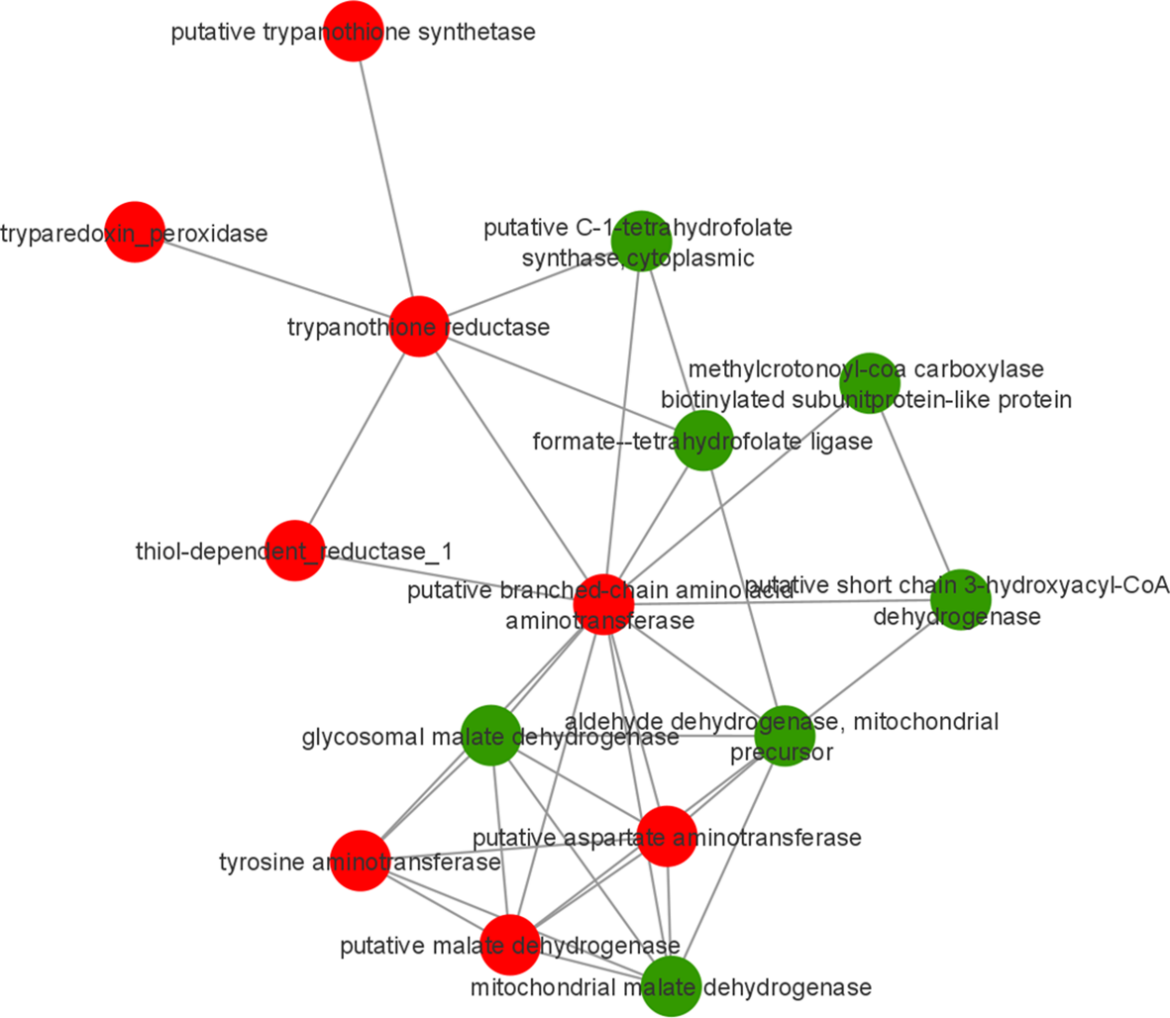
Prediction of interaction among differentially abundant proteins in *L. braziliensis* that are part of enriched pathways. Red and green represents upregulated and downregulated proteins in LbAL *vs* LbTL comparison.

The KEGG tool was used to identify the most relevant pathways associated with regulated proteins in the promastigote forms of *Leishmania*, causing atypical lesions. Regarding positively regulated proteins, the analysis showed the enrichment of five pathways: cysteine and methionine metabolism (*P*=1.58e-4, with 10 proteins), glutathione metabolism (*P*=5.96e-3, with 6 proteins), phenylalanine metabolism (*P*=6.79e-3, with 6 proteins), polyketide sugar unit biosynthesis (*P*=8.46e-3, with 6 proteins), and tyrosine metabolism (*P*=9.85e-3, with 9 proteins). In contrast, negatively regulated proteins participated in six pathways: carbon fixation pathways in prokaryotes (*P*=1.37e-4, with 8 proteins); valine, leucine, and isoleucine degradation (*P*=4.60e-3, with 5 proteins); benzoate degradation (*P*=7.99e-3, with 5 proteins); glyoxylate and dicarboxylate metabolism (p=8.69e-3, with 5 proteins); geraniol degradation (*P*=9.85e-3, with 3 proteins); and one carbon pool by folate (*P*= 9.85e-3, with 3 proteins) (**Figure 4**). Proteins belonging to each pathway are listed in **Table 3**, where it can be observed that each protein participates in more than one pathway, except in the case of glutathione metabolism. To verify the interactions between the identified proteins belonging to the enriched pathways, prediction analysis of protein interactions was performed (**Figure 5**), highlighting that the dehydrogenases were negatively regulated and interacted with each other, and trypanothione and tryparedoxin were positively regulated and interacted with each other.

**Table 3:** KEGG enriched pathways of differentially regulated proteins in *L. braziliensis* from atypical lesion (LbAL) compared to *L. braziliensis* from typical lesion (LbTL).

PATHWAY	PROTEINS	RATIO(LbAL *vs* LbTL)
**UPREGULATED**		
Cysteine and methionine metabolism	putative thymine-7-hydroxylase	1.456151
	**FtsJ-like_methyltransferase**	1.287025
	**putative sterol 24-c-methyltransferase**	0.814614
	putative methylthioadenosine phosphorylase	0.717098
	**conserved hypothetical protein (134063540)**	0.686384
	**putative aspartate aminotransferase**	0.679376
	putative branched-chain amino acid aminotransferase	0.639421
	**tyrosine aminotransferase**	0.627521
	**putative arginine N-methyltransferase, type III**	0.616916
	putative malate dehydrogenase	0.585591
Glutathione metabolism	trypanothione reductase	2.417349
	hypothetical protein, unknown function (322504762)	1.724753
	tryparedoxin_peroxidase	0.993897
	thiol-dependent_reductase_1	0.708709
	putative trypanothione synthetase	0.689969
	putative glutaredoxin-like protein	0.649122
Phenylalanine metabolism	**FtsJ-like_methyltransferase**	1.287025
	**putative sterol 24-c-methyltransferase**	0.814614
	**conserved hypothetical protein (134063540)**	0.686384
	**putative aspartate aminotransferase**	0.679376
	**tyrosine aminotransferase**	0.627521
	conserved hypothetical protein (134063845)	0.598311
Polyketide sugar unit biosynthesis	**FtsJ-like_methyltransferase**	1.28703
	**putative sterol 24-c-methyltransferase**	0.81461
	**conserved hypothetical protein (134063540)**	0.68638
	**tyrosine aminotransferase**	0.62752
	**putative arginine N-methyltransferase, type III**	0.61692
	**putative lanosterol synthase**	0.61456
Tyrosine metabolism	**FtsJ-like_methyltransferase**	1.28703
	**putative sterol 24-c-methyltransferase**	0.81461
	Acyltransferase	0.81126
	putative NADP-dependent alcohol dehydrogenase	0.69938
	**conserved hypothetical protein (134063540)**	0.68638
	**putative aspartate aminotransferase**	0.67938
	**tyrosine aminotransferase**	0.62752
	**putative arginine N-methyltransferase, type III**	0.61692
	**putative lanosterol synthase**	0.61456
**DOWNREGULATED PROTEINS**		
Carbon fixation pathways in prokaryotes	**mitochondrial malate dehydrogenase**	-1.1499
	**glycosomal malate dehydrogenase**	-0.94403
	**putative short chain 3-hydroxyacyl-CoA dehydrogenase**	-0.82537
	formate—tetrahydrofolate ligase	-0.81918
	**methylcrotonoyl-coa carboxylase biotinylated subunitprotein-like protein**	-0.68285
	putative C-1-tetrahydrofolate synthase,cytoplasmic	-0.65468
	**enoyl-CoA hydratase/isomerase family protein,conserved**	-0.62284
	**mitochondrial DNA topoisomerase II**	-0.60924
Valine, leucine and isoleucine degradation	**putative short chain 3-hydroxyacyl-CoA dehydrogenase**	-0.82537
	**aldehyde dehydrogenase, mitochondrial precursor**	-0.69571
	**methylcrotonoyl-coa carboxylase biotinylated subunitprotein-like protein**	-0.68285
	**enoyl-CoA hydratase/isomerase family protein,conserved**	-0.62284
	**mitochondrial DNA topoisomerase II**	-0.60924
Benzoate degradation	**putative short chain 3-hydroxyacyl-CoA dehydrogenase**	-0.82537
	**aldehyde dehydrogenase, mitochondrial precursor**	-0.69571
	**methylcrotonoyl-coa carboxylase biotinylated subunitprotein-like protein**	-0.68285
	**enoyl-CoA hydratase/isomerase family protein,conserved**	-0.62284
	**mitochondrial DNA topoisomerase II**	-0.60924
Glyoxylate and dicarboxylate metabolism	**mitochondrial malate dehydrogenase**	-1.1499
	**glycosomal malate dehydrogenase**	-0.94403
	**methylcrotonoyl-coa carboxylase biotinylated subunitprotein-like protein**	-0.68285
	**enoyl-CoA hydratase/isomerase family protein,conserved**	-0.62284
	**mitochondrial DNA topoisomerase II**	-0.60924
Geraniol degradation	**putative short chain 3-hydroxyacyl-CoA dehydrogenase**	-0.82537
	**aldehyde dehydrogenase, mitochondrial precursor**	-0.69571
	**enoyl-CoA hydratase/isomerase family protein,conserved**	-0.62284
One carbon pool by folate	**putative short chain 3-hydroxyacyl-CoA dehydrogenase**	-0.82537
	**aldehyde dehydrogenase, mitochondrial precursor**	-0.69571
	**enoyl-CoA hydratase/isomerase family protein, conserved**	-0.62284

In bold proteins that are in more than one pathway, proteins sorted by log2-ratio.

### 3.5 Peroxidase Activity

**Figure 6:**
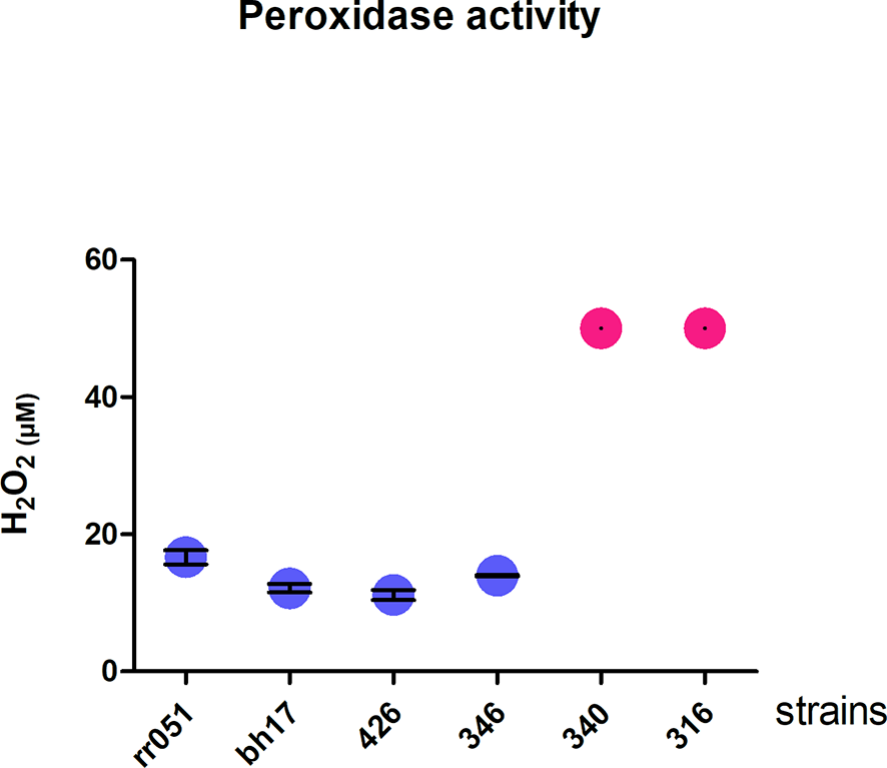
Peroxidase activity. Degradation of hydrogen peroxide by cell lysates of promastigote forms of different strains of *Leishmania braziliensis*. In blue, LbTL group; in pink, LbAL group. These experiments were performed twice in triplicate. Measurements from the atypical group exceeded the test capacity (50µM) and, thus, no standard deviation can be calculated.

Glutathione (GSH) plays a central role in cell defense against oxidative stress. In trypanosomatides, the removal of peroxides is performed by tryparedoxin peroxidase (Collotti et al., 2013). Tryparedoxin peroxidase was found among the upregulated proteins belonging to the glutathione pathway (ratio LbAL ***vs*** LbTL = 0.993897). Therefore, the antioxidant capacities of different strains were evaluated. Since after 30 min of exposure to 50 µM hydrogen peroxide, protein extracts from strains belonging to the atypical group consumed all H_2_O_2_, the reported value should be interpreted as lower boundary (no standard deviation can be calculated), while those in the typical group consumed an average of 15.7 µM of H_2_O_2_. The hydrogen peroxide consumption in the atypical group was 3.01-fold higher than that in the typical group (*P*<0.001; **Figure 6**).

## 4 Discussion

Determining factors of atypical cutaneous leishmaniasis (ACL) caused frequently by *Leishmania (Viannia) braziliensis* remain unclear. It is possible that these clinical forms are determined by host and parasite factors, as demonstrated for other clinical forms of leishmaniasis. However, histopathological analysis of cutaneous tissue samples and cytokine/chemokine expression in skin biopsies from ACL patients showed no association with the type of injury (Costa-Silva et al., 2014). In contrast, the parasite presented genetic polymorphism correlated with atypical clinical manifestations (Quaresma et al., 2018) and was more resistant to N-methylglucamine antimonate (Rugani et al., 2019); clinical cases have been associated with therapeutic failures (Guimarães et al., 2016). Thus, we performed a comparative proteomic analysis to identify differentially abundant proteins among *L. (V.) braziliensis* strains isolated from atypical lesions (LbAL) compared to isolates from typical lesions (LbTL). The strains used in the present study were previously characterized for genetic polymorphism, as well as for resistance to N-methylglucamine antimonate; the typical strains are genetically non-polymorphic and sensitive to glucantime, whereas the atypical strains are genetic polymorphic and resistant to N-methylglucamine antimonate (Rugani et al., 2018; Quaresma et al., 2018).

Since the role of the parasite in a large clinical plasticity of *L. braziliensis* infections is not clear this study aims to contribute to the knowledge about the factors involved in such pleomorphism. We quantified 4,048 proteins, of which 254 and 196 proteins showed increased and decreased abundance, respectively, in atypical strains compared to typical strains. To identify potential factors related to each strain, proteins were categorized into functional groups based onmolecular function (MF) and biological process (BP), followed by prediction analysis of protein interactions (PPI) as well as pathway enrichment analysis using the KEGG tool. The data pointed to upregulated redox activity in atypical strain, among others, the peroxidase activity test validated the findings.

Inside the mammalian host, *Leishmania* lives in the lethal enzymatic environment of macrophage cells, where they deal with the macrophage-generated oxidative stress to survive. Their survival is attributed to a unique redox metabolism with which the parasite has evolved. In the present study, the oxidoreductase activity of proteins that act on sulfur group donors was upregulated in atypical strains compared to typical strains. Our results show that in this process, five proteins were increased, represented mainly by trypanothione reductase (TR), with a higher positive ratio (2.42) followed by peptide methionine sulfoxide reductase-like (Msr) (ratio =1.06). Both TR and Msr are proteins that catalyze reduction reactions and contribute to the antioxidant defenses of these parasites. TR is an enzyme found in trypanosomatides, equivalent to glutathione reductase. This enzyme is a homodimer NADPH-dependent flavoprotein oxidoreductase that participates in central thiol–disulfide exchange reactions as an electron donor in different metabolic pathways, from the synthesis of DNA precursors to oxidant detoxification (Mittal et al., 2005; Colotti et al., 2013). TR neutralizes hydrogen peroxide produced by macrophages during infection through the tryparedoxin/tryparedoxin peroxidase I (TXN/TXNPx) system (Colotti et al., 2013). Therefore, TR inhibition increases the intracellular levels of reactive oxygen species that are lethal to the parasite. Similarly, Msr is a protein that catalyzes the reduction reaction, in this case, reducing oxidized methionine residues. Rodríguez-Veja et al. (2021) showed that strains of *L. braziliensis* related to disseminated leishmaniasis (DL) might also have a greater resistance to oxidative stress, mediated by the increased abundance of trypanothione reductase, and thioredoxin-like fold domain containing protein. Consistently, disruption of the trypanothione reductase gene decreases the *Leishmania* infectivity and its ability to survive within the macrophage. Thus, our results indicate that the parasites that cause atypical lesions present increased abundance of some proteins related to redox homeostasis, which may confer greater survival inside macrophages.

Pentavalent antimony-containing compounds (sodium stibogluconate Pentostam^®^ and N-methyl-glucamine Glucantime^®^) have been the first-line drugs for the treatment of all clinical forms of leishmaniasis since 1940 (Davidson, 1999), and as no human vaccine is in clinical use, chemotherapy relying on pentavalent antimonial still is the most important control for leishmaniasis. The mechanism of action of antimony has not been fully elucidated; however, SbIII (stibogluconate) is purported to generate disturbances in the thiol redox potential of the parasite by inducing the efflux of intracellular thiols and by inhibiting trypanothione reductase, resulting in cell death by oxidative stress (Wyllie et al., 2004). The antimony susceptibility test (EC 50 assay) revealed that *L. braziliensis* lines overexpressing cytosolic tryparedoxin peroxidase (cTXNPx) had a 2-fold increase in resistance to SbIII compared to the untransfected parental line. Furthermore, these clones were more tolerant to exogenous H_2_O_2_ than the untransfected parental line (Moreira and Murta, 2016). Similarly, overexpression of nucleoside diphosphate kinase b and elongation factor 2 results in an increase in SbIII resistance in *L. braziliensis* (Andrade and Murta, 2014). Corroborating these literature data, the results of the present work, in which we used naturally-occurring antimony-resistant strains that cause AL, showed that these strains present upregulated reductase proteins (trypanothione reductase, thioredoxin, peptide methionine sulfoxide reductase-like, thiol-dependent reductase 1, nucleoside diphosphate kinase b, deoxyribose phosphate aldolase, and methyltioadenosine phosphorilase, which interact with each other (**Figure 3**). Some of these proteins also participate in glutathione metabolism (one of the enrichment pathways shown by the KEGG tool). Atypical strains showed 3.01-fold higher peroxidase activity than typical ones (*P*<0.001, **Figure 5**). As already mentioned, the atypical strains used in present study were previously characterized as resistant to N-methylglucamine antimonate (Rugani et al., 2018) then, we believe that, collectively, it was possible to identify the proteins related to antimony resistance and prove the greater peroxidase activity in strains of the typical group. These data shed new light on ACL cases that have been associated with therapeutic failures.

Therefore, regarding MF, we observed enrichment of “Enzyme inhibitor activity” as upregulated terms in atypical strains, including the proteins ecotin, ecotin-like, and protein phosphatase inhibitor 2 (IPP-2). Ecotin is a serine protease inhibitor produced by hundreds of microbial species, including pathogens such as *Leishmania* spp. Parasites encode inhibitors of serine protease (ISP), which inhibits serine proteases of the immune response system and manipulates the host physiological processes to ensure their survival. In *L. major*, ISP targets host serine peptidases and influences the early stages of infection of the mammalian host (Eschenlauer et al., 2009). Alam et al. (2016) identified, purified, and characterized an endogenous ISP from an Indian strain of *L. donovani*, which causes fatal visceral leishmaniasis, and these inhibitors show varying and entirely contrasting efficacies toward serine proteases of its own as well as of higher organisms. To that authors, this indicates that it accelerates disease progression and drives parasite survival by inhibiting the activities of host serine proteases. Subsequently, it was demonstrated that ecotin blocks activation of the complement lectin pathway by inhibiting its key activator enzymes, MASP-1 and MASP-2. Furthermore, by inhibiting MASP-3, ecotin also disrupts the fundamental link between lectin and alternative pathways. Thus, ecotin is a potent, versatile self-defense tool that blocks multiple antimicrobial activities in the serum. These findings suggest that ecotin may be a relevant antimicrobial drug target (Nagy et al., 2019). Thus, our data suggest that ecotin could be a new drug target to be tested in strains that are resistant to antimony, as already mentioned. Our data also showed positive regulation of protein phosphatase inhibitor 2 (IPP-2). Protein phosphorylation and dephosphorylation are well recognized as important processes that regulate multiple physiological mechanisms. To date, there are no reports on IPP-2 from *Leishmania*; nevertheless, it is a protein involved in the regulation of phosphoproteins and regulation of signal transduction that is able to impede the activity of a protein phosphatases, enzymes that hydrolyzes phosphate groups from phosphorylated proteins. Upregulation of phosphatase inhibitors consequently allows higher activity of phosphorylated proteins, which are associated with diverse cellular functions, including glycolysis, motility, mitosis, apoptosis, cell cycle progression, and signal transduction (Qureshi et al., 2019).

A clustering of several ribosomal proteins interacting with each other was observed with BP enrichment of 12 (from 27) ribosomal proteins, of which 9 interact with each other and all of which are positively regulated in strains from atypical lesions. Eukaryotic ribosomes have approximately 70 proteins, 30 of which are present in the smaller sub-unit and 40 in the larger sub-unit (Tsurugi et al., 1978), therefore, suggesting that 12 regulated proteins may not be related to increased ribosomal activity; however, in *Leishmania* spp., ribosome-related proteins have been considered relevant molecules during infection and have been shown to modulate cellular activity and cytokine release (Cordeiro-Da-Silva et al., 2001). Therefore, they are good candidates for vaccination (Iborra et al., 2008; Brito et al., 2017). These ribosomal proteins could be associated with immune response modulation, favorable to the parasites from atypical strains which the disease duration was significantly longer among ACL individual as reported in Guimarães et al. (2016).

Our results also demonstrate negative regulation of the oxidoreductase activity, acting on CH-OH group of donors, as well as acting on a sulfur group of donors, disulfide as an acceptor, groups represented mainly by malate dehydrogenase and choline dehydrogenase in atypical strains compared to typical strains. In this case, the higher negative ratios were malate dehydrogenase (-1.15) and choline dehydrogenase (-1.06). Furthermore, these categories included several downregulated dehydrogenases (malate dehydrogenase, choline dehydrogenase, dehydrogenase-like protein, short chain 3-hydroxyacyl-CoA dehydrogenase, and aldehyde dehydrogenase) that interact with each other (**Figure 3**). Dehydrogenases are oxidoreductase enzymes that catalyze the transfer of hydrogen and a pair of electrons from a reduced substrate, which is oxidized in the reaction, to an electron-receiving molecule, which in turn, is reduced. In higher eukaryotes, mitochondrial MDH (mMDH) is involved in the transfer of reducing equivalents from the cytosol to the mitochondria, which is essential for the activity of the glycolytic pathway in the cytosol. MDH has been demonstrated in *Leishmania* over 20 years ago (Mottram and Coombs, 1985), although the metabolic roles of MDH isozymes in trypanosomatides have not yet been clearly established. Leroux et al. (2006) demonstrated the presence of three MDH isoforms with slightly distinct biochemical properties and different subcellular localizations (mitochondrial and cytosolic) in *Leishmania* spp., and verified that these isozymes are remarkably more abundant in amastigotes than in promastigotes of *L. mexicana*. These authors presumed that the functional and biochemical features of these isozymes reflect the metabolic adaptation to the different nutrient sources that these parasites have to face during their life cycle. In *L. braziliensis*, MDH was inferred from homology; however, consistent with the present work, de Rezende et al. (2017) found MDH in *L. amazonensis*. Similarly, the presence of choline dehydrogenase in *Leishmania* spp. was inferred from homology. Thus, to date, no reports are available for experimental activity data involving malate dehydrogenase and choline dehydrogenase in *Leishmania* spp., and it is possible to speculate the role of these enzymes in relation to the strains studied in the present work.

Another negative regulation observed in atypical strains was related to serine/threonine kinase activity by the putative target proteins of rapamycin (TOR) and kinase 1 protein kinase. Initially, TORs were identified by mutations that confer resistance to a potent antifungal metabolite called rapamycin (Heitman et al., 1991). TOR kinases are key master regulators in eukaryotes, linking environmental conditions, such as nutrient availability and stimuli, to protein synthesis and cell cycle machinery in order to coordinate cell growth and replication. *Leishmania* TOR mutants were unable to survive or replicate in macrophages *in vitro* or to induce pathology or establish infections in mice *in vivo*. The loss of virulence is associated with a defect in acidocalcisome formation, a unique organelle of protozoans that plays an important role in energy metabolism (Madeira da Silva and Beverley, 2010). Based on these data, the downregulation of TOR in atypical strains could confer less virulence to this strain compared to typical strains; however, only one protein is not sufficient to determine virulence. Thus, we cannot infer the role of regulation of this protein in the strains studied. Similarly, the downregulation of kinase 1 protein alone is very difficult to speculate because it belongs to an important family of proteins. A number of studies have explored the role of individual *Leishmania* protein kinases in promastigote survival, metacyclic differentiation, and amastigote replication, as well as their role in infectivity. Baker et al. (2021) identified regulators of differentiation or survival in a systematic functional analysis of *Leishmania* protein kinases. They applied kinome-wide gene deletion and gene tagging in *L. mexicana* promastigotes to define protein kinases with life cycle transition roles. While 162 are dispensable, 44 protein kinase genes are refractory to deletion in promastigotes and are likely core genes required for parasite replication. Therefore, to understand the role of this molecular function and its downregulated proteins, further studies are needed on the strains used in the present work.

The results presented here show that promastigotes from *L. braziliensis* strains isolated from atypical and typical lesions have proteins with different abundances. The comparison between these strains enabled the reproducible identification of several proteins that distinguish the two groups. Strains isolated from atypical forms showed an upregulation of proteins which may confer greater survival inside macrophages, proteins that influence the early stages of infection of the mammalian host (ISP), and proteins associated with resistance to antimony therapy. Moreover, peroxidase activity was 3.01-fold higher in the atypical strains than in the typical strains.

## Data Availability Statement

The datasets presented in this study can be found in online repositories. The names of the repository/repositories and accession number(s) can be found below: https://www.ebi.ac.uk/pride/archive/, PXD029995.

## Author contributions

BE: Data Curation, formal analysis, Investigation, Methodology, Visualization, Writing – Original Draft Preparation. MM-B: conceptualization, Methodology, Supervision, Writing – Review & Editing. VG: Methodology, Writing – Review & Editing TV-B: conceptualization, Methodology, Writing – Review & Editing. ML: conceptualization, Methodology, Writing – Review & Editing. CG: conceptualization, Writing – Review & Editing. PQ: conceptualization, Methodology, Writing – Review & Editing. HA: conceptualization, funding acquisition, Methodology, Project administration, resources, Supervision, Writing – Review & Editing. All authors contributed to the article and approved the submitted version.

## Funding

This research was supported by the Fundação de Amparo à Pesquisa do Estado de Minas Gerais [PPM00129-17], Instituto Nacional de Ciência e Tecnologia de Vacinas [CNPq-573547/2008-4/FAPEMIG/MS-CBB, APQ 00077-09] and Rede Mineira de Biomoléculas [CCBRED00012-14]. The funders had no role in study design, data collection and analysis, decision to publish, or preparation of the manuscript. HA is CNPq fellows (PQ). BE and MM-B are CAPES fellowship. The funders had no role in study design, data collection and analysis, decision to publish, or preparation of the manuscript.

## Conflict of Interest

The authors declare that the research was conducted in the absence of any commercial or financial relationships that could be construed as a potential conflict of interest.

## Publisher’s Note

All claims expressed in this article are solely those of the authors and do not necessarily represent those of their affiliated organizations, or those of the publisher, the editors and the reviewers. Any product that may be evaluated in this article, or claim that may be made by its manufacturer, is not guaranteed or endorsed by the publisher.

## Supplementary Material

Supplementary Table S1 All proteins identified by MS/MS.

Supplementary Table S2 Proteins identified by MS/MS in each run with abundance increased in Atypical (LbAL) strains compared to Typical (LbTL) strains.

Supplementary Table S3 Proteins identified by MS/MS in each run with abundance decreased in Atypical (LbAL) strains compared to Typical (LbTL) strains.
